# Tongue Ties or Fragments Transformed: Making Sense of Similarities and Differences between the Five Largest English-Speaking Jewish Communities

**DOI:** 10.1007/s12397-023-09477-y

**Published:** 2023-03-20

**Authors:** Adina L. Bankier-Karp

**Affiliations:** Australian Centre for Jewish Civilisation, Monash University, Melbourne, Australia

**Keywords:** Jewish community research, Diaspora Jewry, Hierarchical regression, Research methods

## Abstract

The subjects of Jewish identity and Jewish communal vitality, and how they may be conceptualized and measured, are the topics of lively debate among scholars of contemporary Jewry (DellaPergola 2015, 2020; Kosmin 2022; Pew Research Center 2021; Phillips 2022). Complicating matters, there appears to be a disconnect between the broadly accepted claim that comparative analysis yields richer understanding of Jewish communities (Cooperman 2016; Weinfeld 2020) and the reality that the preponderance of that research focuses on discrete communities.

This paper examines the five largest English-speaking Jewish communities in the diaspora: the United States of America (US) (population 6,000,000), Canada (population 393,500), the United Kingdom (UK) (population 292,000), Australia (population 118,000), and South Africa (population 52,000) (DellaPergola 2022). A comparison of the five communities’ levels of Jewish engagement, and the identification of factors shaping these differences, are the main objectives of this paper. The paper first outlines conceptual and methodological issues involved in the study of contemporary Jewry; hierarchical linear modeling is proposed as the suitable statistical approach for this analysis, and ethnocultural and religious capital are promoted as suitable measures for studying Jewish engagement. Secondly, a contextualizing historical and sociodemographic overview of the five communities is presented, highlighting attributes which the communities have in common, and those which differentiate them. Statistical methods are then utilized to develop measures of Jewish capital, and to identify explanatory factors shaping the differences between these five communities in these measures of Jewish capital. To further the research agenda of communal and transnational research, this paper concludes by identifying questions that are unique to the individual communities studied, with a brief exploration of subjects that Jewish communities often neglect to examine and are encouraged to consider. This paper demonstrates the merits of comparative analysis and highlights practical and conceptual implications for future Jewish communal research.

## Introduction

This paper examines the five largest English-speaking Jewish communities in the diaspora: the United States (US) (pop. 6,000,000), Canada (pop. 393,500), United Kingdom (UK) (pop. 292,000), Australia (pop. 118,000), and South Africa (pop. 52,000) (DellaPergola 2022). A comparison of the five communities’ levels of Jewish engagement, and the identification of factors shaping any differences, are the main objectives of this paper, as realized in the following sections. The first section outlines conceptual and methodological issues involved in the study of contemporary Jewry; hierarchical linear modelling is proposed as the suitable statistical approach for the analysis, and ethnocultural and religious capital are promoted as suitable measures for studying Jewish engagement. The next section presents a contextualizing historical and sociodemographic overview of the five communities, highlighting attributes which the communities have in common, and those which differentiate them. Statistical methods are then utilized to develop measures of Jewish capital, and to identify explanatory factors shaping the differences between the five communities in these measures of Jewish capital. In order to further the research agenda of communal and transnational research, this paper concludes by identifying questions which are unique to the individual communities studied, with a brief exploration of subjects that Jewish communities often neglect to examine and are encouraged to consider. This paper demonstrates the merits of comparative analysis and highlights practical and conceptual implications for future Jewish communal research.

## Literature Review

### Conceptual and Methodological Issues

Beyond the lively debates about how contemporary Jewish identity should be defined (DellaPergola 2015; 2020; Kosmin 2022; Pew Research Center 2021; Phillips 2022) are discussions about the meaning of terminology employed to articulate Jewish identification, the most common being cultural, secular, ethnic, or religious (Mayer et al. 2001; Schnoor 2002). Scholars also ponder how to conceptualize and measure personal, familial, and communal engagement, together with how these might illuminate questions regarding Jewish communal vitality. With an increasing dismissal of the relevance of denominational affiliation (Klaff 2006), there is a greater preference for forms of engagement (Aronson et al. 2022). Analysis of Jewish communal engagement is complicated, however, by a disconnect between the broadly accepted claim that comparative analysis yields richer understanding of Jewish communities (Cooperman 2016; Weinfeld 2020) and the reality that the preponderance of Jewish communal research focuses on discrete communities. Lack of coordination between communities means that a rich array of common variables for comparative analysis is not guaranteed. That being said, scholars of communal research articulate their stances on these issues by the definitions they employ, the populations they include, and the ways they measure engagement.

There are many methods that might be utilized for presenting the differences between these Jewish communities, including similarity structure analysis (DellaPergola et al. 2019) and latent class analysis (Aronson et al. 2019). These approaches attempt visually and conceptually to present commonalities in the ways that a group or subgroups respond to a series of questions. Hierarchical linear modeling (HLM) is an ordinary least squares (OLS) regression technique, which may be used to examine variance in a dependent variable when independent variables have varying hierarchical levels (Garson 2013). Moreover, when independent variables are introduced successively in a series of models, it is possible to evaluate whether the effects of certain independent variables are sustained or attenuated with the introduction of others. HLM was therefore deemed suitable for this analysis, since it is not only possible to compare the five Jewish communities on various measures of engagement, but it is also possible to assess theories about which factors might underlie these differences.

### Jewish Ethnocultural and Religious Capital

Several forms of capital are useful for conceptualizing ethnocultural and religious engagement. *Cultural capital* includes the information, skills, and wisdom which are prized by a particular society (Bourdieu 2011). *Social capital* describes the social bonds and networks accrued by individuals as a consequence of socially approved attitudes and conduct (Putnam 2000). Finally, *religious capital* encompasses religious faith and conduct, together with familiarity and fluency with the religion’s ideologies, traditions, and rituals (Iannaccone 1990). Given that ethnocultural forms of Jewish engagement require a degree of familiarity, are performative, and are highly social (Kelman et al. 2017), the concepts of cultural and social capital are both subsumed in the notion of Jewish ethnocultural capital used in this study. Secondly, given the centrality of both religious conviction as well as practical conduct, religious capital informs the notion of Jewish religious capital used in this study.^1^ Higher measures of Jewish ethnocultural and religious capital are defined in this paper as higher levels of Jewish engagement.

### The Five Communities under Investigation

In the opening pages of his classic *The Founding of New Societies*, historian Louis Hartz (1964) made the following observation about the UK and its relationship with the nations of Australia, Canada, South Africa, and the US:

[A]ll of them are fragments of the larger whole of Europe struck off in the course of the revolution which brought the West into the modern world … and hurled outward onto new soil … On the surface it might seem, precisely because the content of many of the other fragment cultures differs so widely from our own, that there could be no possible connection… When a fragment of Europe becomes the whole of a new nation, it becomes unrecognizable in European terms ... First of all, it becomes a universal, sinking beneath the surface of thought to the level of assumption. Then, almost instantly, it is reborn, transformed into a new nationalism arising out of the necessities of fragmentation itself. – Louis Hartz (1964: 3−5)

The five Jewish communities which are the subject of this paper have in common a British origin story. With the passage of time, however, do these communities have enduring commonalities? Mendelsohn (2007) claims common language and continuous communication between Jews in the “Anglophone diaspora… knitted the Jewish communities of the English-speaking countries into a new cultural, religious and social sphere” (p. 178). Despite distance and the passage of time, Mendelsohn argues that the continuous exchange of news, conventions, and styles created a common Anglophone culture. Opposing this proposition is Bronfenbrenner’s Bioecological Development Theory (2001), which claims that human development is subject to the influence of a series of nested and connected settings. Beyond the home, workplace, and local Jewish community institutions, Bronfenbrenner highlights the at-times imperceptible influence of national social structures, cultural and social norms. Such a contention would suggest that, despite intercommunal communication, the nation itself would shape its Jewish community. As Hartz noted in the above extract, the Jews from these five countries may have British origins, but with the passage of time, how much do these Jewish communities have in common? Does Mendelsohn’s (2007) claim about the power of a connected Anglophone find validation in strong similarities across the five communities? Or does Bronfenbrenner’s (2001) argument about the power of the national context find expression in variability vis-à-vis ethnocultural and religious capital? What forms of Jewish expression characterize and distinguish these communities, and what underlies these differences? The following overview traces historical and sociodemographic attributes identified as distinguishing these five communities.

The story of British Jewry is comparatively lengthier and more volatile, commencing when Jews were believed to have first arrived in England with the Norman conquest in 1066 (Scheil 2004), involving persecution and eventual expulsion in 1290, the modern Jewish community in Britain dates from the 1630s with the arrival in London of *conversos* from Spain (Endelman 2002). The first documented Jew to set foot on North American soil was part of the British effort to settle America in 1585, the first congregation founded in New York in 1730 (Sarna 2019). The earliest documented Jews to arrive in Canada were five Jewish soldiers fighting to claim Canada for the British in 1760 during the Seven Years’ War (Rosenberg 1993), the first congregation founded in 1768 in Montreal by British and Dutch Sephardic immigrants (Tulchinsky 1993). The first Jews to arrive in Australia were a dozen Jewish convicts aboard the First Fleet in 1788, the Australian Jewish community being formed in the late 1820s in Sydney with the establishment of a Jewish cemetery and the first Jewish prayer services (Rutland 2006). The South African Jewish community was founded by Anglo-German Jewish settlers in the wake of British occupation of the Cape in 1806 (Shain 2011), the first congregation being founded in 1841 in Cape Town (Herrman 1935).

#### Australia

The Australian Jewish population is estimated to be 117,903, comprising 0.5% of the national population (Graham 2021; Graham and Narunsky 2019), and is the tenth-largest Jewish community in the world (DellaPergola 2022). Australian Jewry is highly concentrated, with 84% living in the cities of Melbourne and Sydney, and over half (51%) living in just four out of Australia’s 557 Local Government Areas, in contrast with 1.5% of the general Australia population (Graham and Narunsky 2019). Australian Jewry is regarded as slightly older when compared with the general Australian population (Graham and Narunsky 2019), and also has a substantial immigrant population, 57% born in other countries (Graham and Markus 2018).

Historical factors that have been highlighted as contributing to Australian Jewry’s strong sense of community include the doubling of the population by the large influx of Holocaust survivors during and just after World War II, community leaders at the time seeing in the aftermath of the Holocaust, assimilation, and antisemitism an urgent need for Jewish day schools (Kipen 2014; Patkin 1972; Rutland 2006). No doubt, chain migration that brought families and friends to the same cities would have further intensified investment in community building (Taft and Markus 2018). It has also been argued that high geographic concentration, together with low geographic mobility, underpins Australian Jewry’s powerful sense of communal identity (Bankier-Karp 2022; forthcoming). A strong sense of community-mindedness is evident in the 70% who reported feeling “very” or “somewhat” connected to Jewish communal life (Graham and Markus 2018). While 52% of Australian Jewry said they felt a strong sense of belonging in Australia and 83% indicated that, in general, people in Australia can be trusted (Graham and Markus 2018), local context should not be ignored. In Australia, multiculturalism is celebrated (Moran 2011), and hybrid identity (Carter 2006) is normative, with comparatively less pressure to assimilate compared with other diaspora communities. This is seen in the overall Australian intermarriage rate of 33% for marriages that occurred in the decade to 2017, a rate that is currently similar to that of the UK (Graham 2018). Australian Jewry is also distinguished by the high proportion who received a Jewish day school education (51%), with 25% who attended for 11 years or more. In addition, Australian Jewry has a distinctive denominational composition, with 23% identifying as Orthodox and only 11% identifying as Reform. A small proportion of Australian Jews identify as Haredi or ultraorthodox (4.4%) (Bankier-Karp 2020), so this small group is unlikely to have exerted a distorting effect on Australian Jewry’s measures of religious capital.

Australian Jews engage to a high degree in what are conventionally understood as religious rituals; the majority of Australian Jews, however, regard these rituals as ethnocultural forms of engagement (Bankier-Karp 2020; Bankier-Karp and Graham forthcoming). This strong degree of ethnocultural engagement is seen in the fact that 92% had visited Israel (77% more than once), 72% had made a financial donation to a Jewish charity in the past 12 months, 82% indicated that all or most of their close friends were Jewish, and 97% read Jewish newspapers or sought out Jewish news online. Religious engagement was embraced, however, by a smaller proportion of Australian Jews, 38% indicating that observing Jewish law is an essential part of what being Jewish means to them, 30% keeping kosher at home and 27% attending Jewish religious services monthly or more, of whom 15% attend weekly or more. While rituals such as attending a Seder or fasting on Yom Kippur are usually considered measures of religious engagement (Aronson et al. 2022), it is interesting that the high proportions of Australians attending a Passover Seder (97%) and fasting on Yom Kippur (81%) were patterns more consistent with their aforementioned measures of ethnocultural engagement, than their measures of religious engagement. The paucity of questions about Jewish cultural life, however, limited the ability to examine Australian ethnocultural identity in more depth in this study.

#### Canada

The Canadian Jewish population is estimated to be 392,000, comprising 1% of the national population (Brym et al. 2020), and is the fourth-largest Jewish community in the world (DellaPergola 2022). Canadian Jewry is also highly concentrated, with 82% living in the cities of Montreal, Toronto, Winnipeg, and Vancouver, and 48% of Canadian Jewry residing in Toronto (Shahar 2020). The Jewish population of Canada is slightly older when contrasted with the general Canadian population (Shahar 2020) and has a small immigrant population, 30% having been born in other countries (Brym et al. 2019).

Most of Canadian Jewry rated their community as an excellent place to live (60%), scholars of Canadian Jewry identifying a high degree of social acceptance (Weinfeld 2020), strong geographic concentration (Anctil 2011), denominational composition, a high proportion of Holocaust survivors, multiculturalism, and Jewish education (Schnoor 2021), together with strong institutions and low rates of antisemitism (Koffman 2020) as underlying the community’s strong sense of belonging. While 80% of Canadian Jews said they had not experienced discrimination—neither due to their religion nor ethnicity or culture (Brym et al. 2019)—and multiculturalism is a strong social norm (Koffman and Weinfeld 2011; Schnoor 2010), the comfortable social and economic position of Canadian Jewry is regarded as exercising an assimilatory force upon the community, with an increase in intermarriage being one consequence (Brym and Lenton 2020). That being said, however, the intermarriage rate in Canada is 26% (Shahar and Schnoor 2015). Forty-four percent of Canadians had received a Jewish day school education, 25% having attended for 10 years or more (Brym et al. 2019). In addition, Canada has a distinctive denominational composition, with 26% identifying as Conservative and only 16% identifying as Reform. A small proportion of Canadian Jews identify as Haredi, one recent report estimating that this group constitutes 8% of Canadian Jewry (Staetsky 2022). As is the case in Australia, it is therefore unlikely that the Haredi population substantially affected Canadian Jewry’s measures of religious capital.

The basis of Jewish identity in Canada is more about ethnocultural forms of engagement than those involving religion (Brym et al. 2020; Cappucci 2021a; Shahar 2020). The greater focus on ethnocultural engagement is also seen in the fact that 73% had visited Israel (53% more than once), 85% made a financial donation to a Jewish charity in the past 12 months, and 61% said all or most of their close friends were Jewish, with a smaller 29% reading Jewish newspapers or seeking out Jewish news online. Religious engagement was embraced by a smaller proportion of Canadian Jews, 18% indicating that observing Jewish law is an essential part of what being Jewish means to them, and 30% attending Jewish religious services monthly or more, of whom 16% attended weekly or more. In addition, 32% said religion was very important in their lives (70% saying it was very or somewhat important). In a qualitative study of the Canadian community of Windsor-Essex, it was argued that kosher laws are regarded as markers of religious adherence (Cappucci 2021b). The absence, however, of questions about attendance at a Passover Seder, fasting on Yom Kippur, or observance of kosher rules in the most recent Canadian Jewish community survey prevented the determination of whether these rituals were observed in frequencies more similar to their patterns of ethnocultural or religious engagement.

#### South Africa

South Africa’s Jewish population is estimated at 52,000, comprising 0.09% of the national population (Graham 2020), and is the 11th-largest Jewish community in the world (DellaPergola 2022). South African Jewry is highly concentrated, with 82% living in the cities of Johannesburg and Cape Town, 58% living in Johannesburg (Graham 2020). The Jewish population of South Africa is slightly older when contrasted with similar populations such as Australia and the UK. In addition, South Africa has a comparatively small immigrant population, 11% born in other countries (Graham 2020. For the three previous community studies, see Bruk 2006, DellaPergola and Dubb 1988, and Dubb 1994).

The particular sociohistorical circumstances of South African Jewry encouraged Jewish particularism; namely, the reality of living among the highly divided white Afrikaners, during and in the aftermath of the institutionalized racial segregation policies impacting upon the Black majority (Shain 2011). These turbulent conditions have fostered a sense of trepidation in the South African Jewish community, which has coincided with this cohesive community’s comparatively higher ethnocultural and religious forms of engagement (Kosmin et al. 1999). Indeed Tatz et al. (2007) maintain that “faith is an antidote to fear” (p. 126), the South African community’s comparably high level of religiosity being attributed to the country’s political and economic instability. It has been argued (Beider 2022) that an even more complex constellation of factors is at play, differential fertility patterns, migration, the political–economic context, as well as religious switching—in particular due to secularization on the one hand and, on the other, to the success of *kiruv* (religious outreach) organizations such as Ohr Somayach (Fachler 2022)—causing a shrinking of the community’s center, which may amplify both the effects of an increased religiosity as well as secularity. Despite three-quarters (74%) of South African Jews indicating that they had a very strong or quite strong sense of belonging to South Africa, over one-third (37%) reported their intention to leave their current location within the next 5 years, 41% of whom indicating they intended to leave South Africa. Of those intending to leave South Africa, over half (51%) indicated they would move to Israel, half of those due to concerns about the future of South Africa and fear for their personal safety. The intermarriage rate in South Africa for those who married in the 5 years prior to the community study was 19% (Graham 2020). Forty-five percent of South Africans received a Jewish day school education. In addition, South Africa has a distinctive denominational composition, 30% identifying as Orthodox and 32% as Traditional (Graham 2020). Denomination has long been criticized as a marker of Jewish identity (Klaff 2006), in part due to the highly localized meaning of denominational terminology. South African Jews have been described as favoring “non-observant Orthodoxy” (Hellig 1987), which partly makes sense of the higher salience of ethnocultural rather than religious forms of engagement, given that the former measures are attractive to both the highly and moderately engaged. The proportion of South African Jews who identified as Haredi (8.4%) (Graham 2020), Orthodox, and Traditional may therefore have exercised an effect on measures of religious capital.

The basis of Jewish identity in South Africa is a strong combination of religious and ethnocultural forms of engagement (Graham 2020), with noticeable effects of the religious revival in the country’s recent history (Shain 2021). The importance, however, of ethnocultural engagement is seen in the fact that 89% have visited Israel (75% more than once), 100% made a financial donation to a Jewish charity in the past 12 months, and 85% said all or most of their close friends were Jewish, with 97% reading Jewish newspapers or seeking out Jewish news online. In addition, 67% felt accepted in the Jewish community. Religious engagement is embraced by a smaller but not insignificant proportion of South African Jews, 24% indicating that observing Jewish law was an essential part of what being Jewish means to them, 33% eating only kosher meat at home, and 49% attending Jewish religious services monthly or more, of whom 33% attended weekly or more. That 81% held or attended a Passover Seder and 91% fasted on Yom Kippur (Graham 2020) reveals patterns in these measures which are more consistent with South African Jewry’s ethnocultural rather than religious measures of engagement. As was the case in Australia, Jewish culture was not as comprehensively covered in the South African Jewish community study, preventing a more in-depth exploration of the effects of political instability on ethnocultural engagement.

#### United Kingdom

The Jewish population of the UK is estimated to be 292,000, comprising 0.44% of the national population (JPR 2022), and is the fifth-largest Jewish community in the world (DellaPergola 2022). British Jewry is also highly concentrated, with three-quarters living in Greater London and the Southeast of England (Graham et al. 2014). The Jewish population of the UK is older when contrasted with the general British population and has a small immigrant population, 23% born in other countries (Graham et al. 2014).

Multiculturalism may be normative in the UK (Ashcroft and Bevir 2018), but the increasing secularization of the majority reveals the extent to which assimilatory forces are impacting upon the community, with changes to Jewish philanthropy being one consequence (Graham and Boyd 2016). In addition, the intermarriage rate in the UK for marriages in the 5 years prior to 2014 is 25% (Graham et al. 2014). Thirty percent of UK Jews received a Jewish day school education, with the proportions of those receiving a day school education having increased steadily for several decades (Horup et al. 2021). In addition, the UK has a distinctive demographic composition, with 16% identifying as Orthodox and 26% identifying as Traditional. A small proportion of British Jews identified as Haredi (3.7%) (Graham et al. 2014), this community’s high birth rate being argued to have and continue exercizing influence on British Jewry’s measures of religious capital (Staetsky 2022). As is the case in South Africa, the fracturing of the community into increasingly large proportions of highly secular and highly religious Jews may distort aggregated community snapshots.

The bases of Jewish identity in the UK are predominantly ethnocultural forms of engagement, with identification primarily in terms of culture and ancestry (DellaPergola and Staetsky 2021). The importance of ethnocultural engagement is seen in the fact that 93% made a financial donation to a Jewish charity in the past 12 months, 70% said all or most of their close friends are Jewish, and 92% read Jewish newspapers or sought out Jewish news online. Religious engagement was embraced by a smaller proportion of British Jews, with 38% indicating that observing Jewish law was an essential part of what being Jewish meant to them, 48% eating only kosher meat at home, and 46% attending Jewish religious services monthly or more, of whom 28% attended weekly or more. That 92% held or attended a Passover Seder and 76% fasted on Yom Kippur (Graham et al. 2014) also reveals patterns that are more consistent with UK Jewry’s ethnocultural rather than religious measures of engagement. The UK survey did not include a question about the frequency and duration of Israel visits—questions relating to participation in particular Israel programs such as Birthright being insufficiently similar for comparative analysis. Sixty-nine percent of UK Jews, however, indicated that supporting Israel was very or fairly important to their sense of Jewish identity, suggesting a high degree of Israel attachment.

#### US

The Jewish population of the US is estimated to be between 6,000,000 (DellaPergola 2022) and 7,600,000 (Saxe et al. 2021), comprising 2–2.4% of the national population (Saxe et al. 2021), and is home to the second-largest Jewish community in the world (DellaPergola 2022). US Jewry is also fairly concentrated, with half living in the three states of New York, California, and Florida, 25% of US Jewry living in the New York–Newark–Jersey City metro area (Saxe et al. 2021). The Jewish population of the US is disproportionately older when contrasted with the general US population and has a small immigrant population, 9% having been born in other countries (Pew Research Center 2021).

In a country where secularization, religious exogamy, and racial and ethnic diversity are on the rise (Pew Research Center 2021), Jews are highly socially integrated (Kadushin et al. 2012). These social forces influence US Jewry, the intermarriage rate in the US for marriages in the decade prior to 2021 is 60% (Pew Research Center 2021). Fifteen percent of US Jews received a Jewish day school education, close to 8% receiving 7 years or more of Jewish schooling (Pew Research Center 2021). In addition, the US has a distinctive demographic composition, with 31% identifying as Reform and close to half (47%) having no denomination. A small proportion of US Jewry identify as Haredi (3%) (Pew Research Center 2021), this small population unlikely to have influenced US Jewry’s measures of religious capital as much as the majority that reported no denomination.

The bases of Jewish identity in the US are predominantly ethnocultural and secular forms of engagement (Pew Research Center 2021). The salience of ethnocultural engagement is seen in the fact that 33% had visited Israel (17% more than once), 35% made a financial donation to a Jewish charity in the past 12 months, 20% said all or most of their close friends were Jewish, and 57% read Jewish newspapers or sought out Jewish news online. Religious engagement appears to be embraced by a smaller proportion of US Jews, with 15% indicating that observing Jewish law was an essential part of what being Jewish means to them, 15% keeping kosher at home, and 15% attending Jewish religious services monthly or more, of whom 9% attended weekly or more. Consistent with patterns observed in the other four countries, despite rituals such as attending a Seder and fasting on Yom Kippur being conventionally regarded as measures of religious engagement, US Jewry’s proportions attending a Seder (46%) and fasting on Yom Kippur (40%) (Pew Research Center 2021) manifest patterns that are more similar to their aforementioned measures of ethnocultural engagement compared with their measures of religious engagement. The Pew survey contained the richest variety of cultural measures of the five datasets. Given high geographic mobility, however, the lack of questions about length of dwelling in their local Jewish community prevented examination of the relationship between this factor and a sense of community connectedness.

This historical and sociodemographic overview identified several similarities between the five communities, including a British origin story, higher mean age than the national population, post-World War II immigration intake which included Holocaust survivors (perhaps with the exception of South Africa), and small Haredi populations. Differences between these communities, however, abound. These include the Jewish communities’ age, population size, percentage of the national population, rates of intermarriage, Jewish education, and denominational composition. Patterns in questions about ethnocultural and religious forms of engagement were clear to the extent that South African Jews appeared most highly engaged, Australian, Canadian, and UK Jews were slightly less engaged, and US Jews were the least engaged across the reported variables. The local effects of history, geography, economics, politics, and many more factors may have exercised effects on the communities which are not visible in an initial analysis.

This paper will now examine the Jewish engagement of the Jewish communities of Australia, Canada, South Africa, the UK, and the US. This will be done by comparing their forms of ethnocultural and religious capital, together with what factors might underlie any variability that distinguishes them.

## Methodology

### Datasets

This study uses five datasets which are the most recent national community studies for the five largest English-speaking Jewish diaspora communities (Appendix Table 4). The five datasets may be compared as each was presented as a representative sample of the Jews in that country. Each dataset came with weights to make the sample representative of the larger population being studied (i.e., by age, sex, and geographical location); these weights are used in this analysis.^2^ For the estimation of variance, the combined dataset is analyzed as being composed of five strata, with just under 25,000 individuals sampled from the strata. In the Australian, Canadian, South African, and UK community studies, eligibility was predicated on positive responses to the screener question inquiring whether they regarded themselves as “Jewish in any way.” Positive responses to a series of screener questions in the US community study categorized self-identifying Jews as either “Jews by religion” (*n* = 3836) or “Jews of no religion” (*n* = 882). Other respondents not identifying as Jewish were categorized as people of “Jewish background” (*n* = 802) or people of “Jewish affinity” (*n* = 361). For comparability between the five community studies, only the former self-identifying US Jews were included in this analysis.

### Hypotheses

The hypotheses are that countries will have higher levels of Jewish capital (ethnocultural and religious) if they have higher proportions of people who:

#### Hypothesis 1:

are migrants. This is grounded in research identifying countries with high proportions of immigrants as having higher levels of Jewish engagement (Bankier-Karp 2022).

#### Hypothesis 2:

received a Jewish education. This is grounded in extensive research which identifies strong relationships between Jewish education, broadly construed, and higher levels of Jewish engagement (Boxer 2014).

#### Hypothesis 3:

are endogamously married or partnered. This is grounded in extensive research revealing that endogamously married or partnered people have higher levels of Jewish engagement than those married or partnered exogamously (Cohen 2012; DellaPergola 2011; Hartman 2017; Hartman and Hartman 2009).

#### Hypothesis 4:

have children in their homes. This is grounded in research showing that people raising children have increased Jewish engagement (Aronson et al. 2022; Boxer et al. 2020; Goldscheider 1973).

### Measures

Variables were selected for this analysis where they were either identically worded in the five surveys or could be used as reasonable proxies for the phenomenon being measured.

#### Dependent Variables

A factor analysis with oblique rotation (direct oblimin) resulted in two factors with loadings above 0.4 which, in combination, explained 54% of the variance (Table 1). The first of these factors represents ethnocultural capital (Cronbach’s *α* = 0.70), and the second represents religious capital (Cronbach’s *α* = 0.75), both of which were used as dependent variables in this study.

**Table 1:** Summary of the Factor analysis of nine items measuring Jewish engagement (Pattern Matrix)

	Rotated factor loadings	
Items	Ethnocultural capital factor	Religious capital factor
Last Passover, did you hold or attend a Seder?	**0.72**	−0.05
How many times, if ever, have you been to Israel?	**0.68**	0.22
How many of your close friends are Jewish?	**0.61**	−0.15
Read Jewish newspapers or seek out Jewish news online	**0.60**	−0.02
Financial donation to any Jewish charity or cause in past 12 months	**0.59**	−0.11
During the last Yom Kippur, did you fast?	**0.55**	−0.35
How important is observing Jewish law to what being Jewish means to you?	−0.08	**−0.90**
Do you keep kosher in your home?	0.03	**−0.81**
Frequency of attendance of Jewish religious services at a Jewish congregation	0.21	**−0.73**
Eigenvalues % Variance explained Cronbach’s *α*	3.678 41% 0.70	1.214 13% 0.75

Factor loadings > 0.40 are in boldface. While there were many more questions in the combined dataset relating to forms of Jewish capital, they were omitted from the analysis due to lack of variance

#### Independent variables of interest

The independent variables of interest measured: age, sex, immigrant status, Jewish educational background, relationship status, and child-rearing status. The effects of age (Shulgin et al. 2019) and sex (DellaPergola 2020) on religiosity have been identified, their inclusion allowing the effects of the other independent variables to be tested. Table 2 details descriptive statistics for the study variables.

**Table 2:** Descriptive statistics for the study variables

Variables	M	SD	Actual range	Possible range	Sample size
*Dependent variables*					
Ethnocultural capital factor^a^	5.96	1.77	0–8	0–8	23,600
Religious capital factor^b^	2.55	1.92	0–6	0.6	23,584
*Independent variables*					
Country^c^	–	–	1–5	1–5	23,603
Age^d^	49	0.50	1–4	1–4	23,497
Sex^e^	0.53	0.50	0–1	0–1	23,536
Immigrant status^f^	0.71	0.45	0–1	0–1	22,310
Jewish educational background^g^	1.82	0.39	0–2	0–2	23,563
Relationship status^h^	2.55	0.70	1–3	1–3	22,550
Childrearing status^i^	0.27	0.44	0–1	0–1	20,611

^a^ *Ethnocultural capital scale*: “Last Passover, did you hold or attend a Seder?” (*No* = 0, *Yes* = 1), “How many times, if ever, have you been to Israel?” (*Never* = 0, *Once* = 1, *More than once* = 2), “Have you made a financial donation to any Jewish charity or cause in past 12 months?” (*No* = 0, *Yes* = 1), “How many of your close friends are Jewish?” (*None* = 0, *Some of them* = 1, *All, or most of them* = 2), “Do you read Jewish newspapers or seek out Jewish news online?” (*No* = 0, *Yes* = 1), “During the last Yom Kippur, did you fast?” (*No* = 0, *Yes* = 1)

^b^ *Religious capital scale*: “How important is observing Jewish law to what being Jewish means to you?” (*Not an important part of what being Jewish means to me* = 0, *Important but not essential* = 1, *Essential part of what being Jewish means to me* = 2), “Do you keep kosher in your home?” (*No* = 0, *Yes* = 1), “Frequency of attendance of Jewish religious services at a Jewish congregation” (*Never* = 0, *Seldom/A few times a year* = 1, *Monthly* = 2, *Weekly or more than weekly* = 3)

^c^ *Country* (*Australia* = 1, *Canada* = 2, *South Africa* = 3, *UK* = 4, *US* = 5)

^d^ *Age*: “What is your age?” (*18–29* = 1, *30–49* = 2, *50–64* = 3, *65* +  = 4)

^e^ *Sex*: “What is your sex?” (*Male* = 0, *Female* = 1)

^f^ *Immigrant status*: ‘In what country were you born?’ (*Not an immigrant* = 0, *Immigrant* = 1)

^g^ *Jewish educational background scale*: “When you were growing up, did you receive a Jewish supplemental or day school education?” (*No Jewish education* = 0, *At least Jewish day school education* = *1*). At least Jewish day school education may also include supplemental education. Not all of the datasets contained information on the number of years of day school and supplementary education—in some surveys, these were binary variables. Given that it is unclear for how many years of these forms of education were received, they were combined

^h^ *Relationship status*: “Are you married/in a long-term partnership or de-facto relationship? If so, please describe the religion of your spouse.” (*Married, spouse not Jewish* = 0, *Not married* = 1, *Married, spouse Jewish* = 2). This coding of the relationship status variable was informed by cross-tabulations by relationship status, which revealed consistent patterns with inmarried having the highest, single people having the intermediate, and intermarried people having the lowest levels of ethnocultural and religious capital (Appendix Tables 5 and 6)

^i^ *Childrearing status*: (*No child/ren under 18 in the household* = 0, *Child/ren under 18 in the household* = 1)

#### Analytic Strategy

The five communities were compared across the two engagement scales, which revealed small but statistically significant between-country differences.

Six linear regression models were then used to determine what might account for differences in the two Jewish capital scales—ethnocultural and religious capital—between the five countries. The models examine the association between country and the two scales controlling for age, sex, immigrant status, Jewish educational background, relationship status, and childrearing status. This modeling was conducted to determine whether controlling the independent variables had a statistically significant effect on the coefficients of the country variables—in other words, whether differences between the five countries diminish when controlling for any of the hypothesized predictors of Jewish ethnocultural or religious capital.

### Analysis

The means of the five Jewish communities were compared for their Jewish ethnocultural and religious capital. In all cases, the countries were significantly different from one another (see Appendix Table 7), also confirmed by Games–Howell post-hoc testing (Appendix Tables 8 and 9). While the results for the countries’ measures of Jewish ethnocultural and religious capital were different, they were not entirely inconsistent, with South Africa on the first tier, Australia, Canada, and the UK on the second tier, and the US on the third tier.

#### Explaining the Differences in Ethnocultural and Religious Capital

A three-model HLM was used to analyze the differences between the five communities and which of the four hypothesized variables explained those differences (Table 3). They revealed that the independent variables of interest partly explain differences between the countries in Jewish ethnocultural and religious capital.

**Table 3:** Three-model hierarchical regression, ethnocultural and religious capital

	Ethnocultural capital			Religious capital		
	Restricted model 1	Unrestricted model 2	Unrestricted model 3	Restricted model 1	Unrestricted model 2	Unrestricted model 3
*Country*						
Australia	−0.45*** (0.03)	−0.97*** (0.09)	−0.72*** (0.10)	−0.85*** (0.04)	−1.01***	−0.77***
					(0.08)	(0.10)
Canada	−1.44*** (0.05)	−1.68*** (0.06)	-1.41*** (0.07)	−0.54*** (0.05)	−0.62*** (0.06)	−0.48*** (0.08)
UK	−1.19*** (0.05)	-1.30***	−1.16***	0.11	0.40***	0.57***
		(0.05)	(0.06)	(0.06)	(0.07)	(0.08)
US	−2.56*** (0.07)	−2.31*** (0.07)	−1.60*** (0.08)	−1.05*** (0.06)	−0.96*** (0.06)	−0.52*** (0.09)
Age	0.19* (0.07)	0.15 (0.06)	0.08 (0.06)	-0.07 (0.05)	-0.08 (0.05)	-0.08 (0.05)
*Gender*						
Female	0.21 (0.13)	0.44* (0.13)	0.42*** (0.11)	0.10 (0.10)	0.18 (0.11)	0.15 (0.10)
*Immigrant status*						
Migrant		1.14*** (0.19)	0.82*** (0.18)		0.35* (0.16)	0.15 (0.15)
*Jewish education*						
Jewish day school (or both)		1.67*** (0.13)	1.33*** (0.11)		.68*** (0.12)	0.49*** (0.11)
*Relationship status*						
Inmarried			1.43*** (0.16)			0.60*** (0.13)
Intermarried			-0.80*** (0.15)			-0.91*** (0.12)
*Child rearing status*						
Child/ren in the household			0.38** (0.15)			0.44*** (0.12)
_constant	6.14*** (0.20)	4.65*** (0.21)	3.99*** (0.24)	2.99*** (0.15)	2.41*** (0.20)	2.03*** (0.25)

Note: * *p* < 0.05; ** *p* < 0.01; ***p < 0.001

Omitted categories: South Africa; male; not a migrant; no Jewish education; single; no child/ren in the household. Age was a four-category variable; all categories were included in the above models

**Hypothesis 1**—that countries with larger proportions of migrants will have increased levels of Jewish capital—was partly supported. Having larger proportions of immigrants is significantly and positively associated with Jewish ethnocultural capital, independent of the other predictors in the models. The consistency between higher levels of ethnocultural capital and immigrants suggests that the former is supported by the latter. The fact that Australia has the highest proportion of immigrants partly accounts for these differences. Contrary to hypothesis 1, however, higher proportions of immigrants are not significantly associated with Jewish religious capital.

**Hypothesis 2**—that countries with larger proportions of people who received a Jewish education will have higher levels of Jewish capital—was fully supported. Having larger proportions of people who received a Jewish education is significantly and *positively* associated with Jewish ethnocultural and religious capital. The fact that countries such as the US have fewer respondents who received—and more respondents who did not receive—a Jewish education partly accounts for these differences.

**Hypothesis 3**—that countries with higher proportions of endogamously married and smaller proportions of exogamously married people will have higher levels of Jewish capital—was fully supported. Having larger proportions of people with Jewish spouses or partners is significantly and *positively* associated with Jewish ethnocultural and religious capital. Relatedly, having larger proportions of people with non-Jewish spouses or partners is significantly and *negatively* associated with Jewish ethnocultural and religious capital. The fact that countries such as South Africa have such high proportions of endogamously married people partly explains this difference.

**Hypothesis 4**—that countries with higher proportions raising children under 18 years of age will have higher levels of Jewish capital—is fully supported. Having larger proportions of people with children in their homes is significantly and *positively* associated with Jewish ethnocultural and religious capital. The fact that countries such as Canada have such high proportions of households with children partly explains these differences.

## Discussion

This paper sought to contribute to the scholarship of contemporary Jewry focusing on the largest five English-speaking diaspora Jewish communities and probing questions of Jewish communal vitality by examining Jewish engagement. In addition, the paper sought to identify reasons for the differences between their levels of Jewish engagement. While the literature review included references to historical as well as sociodemographic factors, the chosen approach for this analysis was focusing on common survey items in their most recent community studies. As such, this discussion is mostly confined to the results of this analysis. Jewish engagement was conceptualized as ethnocultural and religious capital, two factors derived from factor analysis. Four hypotheses were proposed as explaining the variance in ethnocultural and religious capital in the five Jewish communities. These hypotheses were that higher proportions of people who are migrants (hyp1), who received a Jewish education (hyp2), who are inmarried (hyp3), and who have children under 18 living with them in their homes (hyp4) will explain higher levels of ethnocultural and religious capital and thus the basis for the intercommunal differences.

Hierarchical linear modeling (HLM) was used to examine the variance in Jewish engagement of the five largest English-speaking Jewish communities. HLM was selected for its capacity to concurrently investigate relationships within and between hierarchical levels of categorized data, and to compute variance among variables at different levels, taking into account the effects of variables at a range of levels on an outcome of interest (Garson 2013).

The between-country differences in levels of ethnocultural capital are attributed to their respective proportions of migrants, those who received a Jewish education, married exogamously, and had children ages 18 and younger living in their homes. The effects of migrants and Jewish education on ethnocultural capital were slightly attenuated with the introduction of the other hypothesized predictors, however, their positive effects remained significant. The largest significant positive effect was endogamous marriage, and relatedly, the largest significant negative effect was exogamous marriage. Nonetheless, Jewish education and having children in the home also exerted significant positive effects. It is worth noting that gender continued to exercise a significant positive effect even with the introduction of all of the hypothesized predictors. It is possible that females contribute more meaningfully than males to the observance of the variables in the ethnocultural capital scale.

Differences in levels of Jewish religious capital may be explained in part by higher proportions who received a Jewish education, the endogamously married, and those with children at home, but not immigrants. The effect of Jewish education attenuated slightly with the introduction of the other hypothesized predictors, however, the positive effect remained significant. Interestingly, the variance explained by having children in the household is highly similar (in terms of the size of the beta coefficients) for both ethnocultural and religious capital; the positive effects of Jewish education and endogamy, and the negative effects of exogamy, have less of an effect on the countries’ levels of religious capital. Perhaps the fact that the majority across all countries are not Orthodox, and Haredim make up only a small percentage of each country, may account for the smaller effects on religious capital.

Owing to the way that HLM is conducted, reference categories are designated (i.e., South Africa, males, and unmarried people), others are compared with them in the analysis, and therefore their effects are not fully understood. Future studies may examine these variables differently, with different reference categories selected to shed further light on the role these independent variables may play in explaining differences in Jewish engagement across these Jewish communities. Owing to the limited number of variables common to the five community studies, this analysis was somewhat limited. The greater methodological flexibility of similarity structure analysis, which caters for not-entirely-or-necessarily-homogeneous sets of variables (DellaPergola et al. 2019) constitutes an attractive alternative, where the desire exists to extend the range of variables included in transnational research.

### A Research Agenda for More Fully Understanding Jewish Engagement in Different Contexts

One of the important practices concerning Jewish community studies is ensuring comparison is possible with other studies (Cooperman 2016; Sheskin 2016). Granted, survey fatigue and drop-out rates make it important for researchers to keep the time burden as low as possible. Moreover, given the often highly localized concerns of communal leadership, even questions with no broader relevance will correctly occupy precious real estate in survey research. However, comparative work—within and beyond the country of concern—benefits when there is a base of shared questions enabling comparative research. To this end, investigators leading future community studies would benefit from coordination and collaboration with international colleagues.

This list is not exhaustive, but it addresses omissions in the community studies that limited comparisons between these countries.

- Knowledge of Hebrew and Yiddish - understanding, speaking, reading, and writing; also, the languages in which people are most fluent and speak at home;
- Antisemitism - how and where it is experienced and how it impacts on Jewish engagement;
- Israel - reasons for connection and disconnection, and how to find ways to bring people together without entirely excluding Israel from the conversation (Kelman and Baron 2019). Few surveys differentiated between Israel programs of different lengths and explored an “immersiveness factor”; namely, the intensity in duration, ideology, and educational content of different programs (Graham 2014);
- Jewish culture - questions that more broadly capture forms of cultural engagement. Examples include naming a Jewish book/film/song/food experience enjoyed recently, and open-ended questions about forms of Jewish culture that people engage in as an expression of their identity;
- Upbringing and the Jewish home - questions about the homes in which respondents grew up and their Jewish background experiences, rather than simply participation in institutional programs. Some contend that the family really matters (Hartman 2020), while some contend that it has ceased to matter (Kelner 2015). The salience, strength, and meaning of those relationships, and how they contribute to Jewish self-understanding and engagement;
- Jewish friends - questions that explore the duration of those friendships and what people do with those friends, trying to more meaningfully tap in on what friends *do* for Jewish socialization. As Kelner argued, “We are who we are by virtue of our relationships” (Kelner 2015: p. 149);
- Subgroup differences - questions that are more sensitive to differences that may exist due to gender, parenting, economics, and geography, for example, using synagogue membership as a proxy for understanding prayer ignores all other potential barriers to synagogue participation, such as membership dues, distance from home, etcetera, which are not necessarily synonymous with obstacles to prayer or engagement with other programmatic offerings;
- COVID-19 - anecdotal evidence suggests that the pandemic may have fundamentally—and irreversibly—changed the ways that individuals, families, and communities engage. To understand people’s experience of loss, fear, isolation and illness, or joy, support, resilience, and revival, community studies could include more open-ended questions to learn about people’s COVID-19 experiences and how organizations can continue offering meaningful opportunities for connection. Mental health and human flourishing scales may also be added, to assist community organizations in understanding Jewish well-being.

## Conclusion

The five Jewish communities examined in this paper have a common British origin story, with Mendelsohn’s (2007) claims about the enduring existence of a common Anglophone culture and Bronfenbrenner’s (2001) claims about effects of different national contexts both supported somewhat in the patterns of engagement across the communities. For while the countries’ measures of Jewish ethnocultural and religious capital were statistically different, they were not entirely inconsistent, South Africa had the highest measures of both forms of capital; Australia, Canada, and the UK inhabited a middle position, and the US had the lowest measures of both ethnocultural and religious capital.

The implications of this analysis are modest but important. Migration status might contribute positively to ethnocultural capital, but mass immigration (outside of Israel) is not a matter for communal policy. Having children at home is also limited in effect, given that having children at home is a limited stage, in the scheme of a full life. However Jewish education has the potential to lead to more informed and inspired Jewish self-identification and Jewish engagement, enriching the experiences of individuals and their loved ones. Increasing access to Jewish education—for children and their families—is a feasible policy with potentially positive outcomes. Finally, with increased access to forms of education and communal engagement, exogamously married couples and their families have the opportunity to become informed, equipped, and inspired to live richer Jewish lives.

## Figures and Tables

**Table 5:**
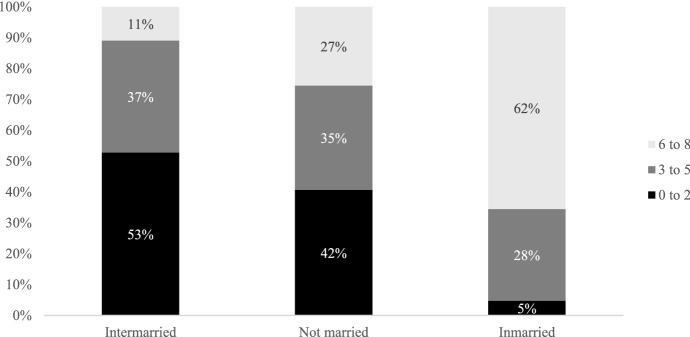
Ethnocultural capital by relationship status

**Table 6:**
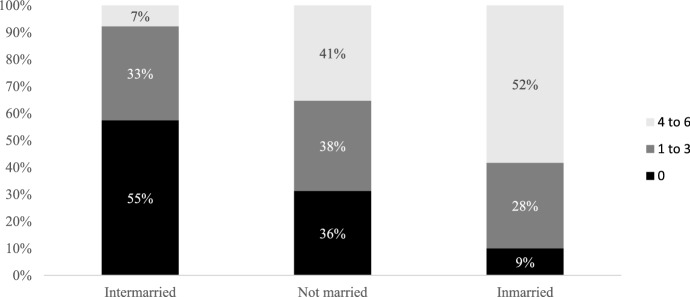
Religious capital by relationship status

## Appendix

See Tables 4

**Table 4:** Datasets used in this analysis

Country and dataset	Research center	Field period	Self-identifying Jewish adults (*N*) in sample	Recruitment method and method of survey completion	Post-hoc weights
Australia *Gen17 Australian Jewish Community Study (2018)*	Australian Centre for Jewish Civilisation, Monash University Melbourne, and JCA, Sydney, Australia	2 February to 22 May 2017	8621	Recruited via community organization member lists, referral, and an open web survey. Completed online	Developed using the Australian Census (2016) religious identity and demographic benchmarks (for age, sex, and state/territory), as well as detailed synagogue membership data*
Canada *The Survey of Jews in Canada (2018)*	Environics Institute for Survey Research, University of Toronto, and York University, Canada	10 February to 30 September 2018	2335	Recruitment informed by census data and distinctive Jewish name lists, in cities with large Jewish populations (i.e., 13,000+), and snowball sampling. Completed online and via telephone	Developed using national census religious identity and demographic benchmarks
South Africa *The Jewish Community Survey of South Africa 2019* (2020)	Institute for Jewish Policy Research, London, UK and Kaplan Centre for Jewish Studies and Research at the University of Cape Town, Australia	9 May to 26 July 2019	4193	Recruitment via community organization member lists, referral, and an open web survey. Completed online	Developed using the Community Survey 2016 conducted by Statistics South Africa, for demographic benchmarks (for age, sex, and state/territory), as well as detailed synagogue membership data^1^
UK *The National Community Survey 2012 (2013)*	Institute for Jewish Policy Research, London, UK	6 June to 15 July 2013	3736	Recruited via community organization member lists, referral, and an open web survey. Completed online	Developed using national census demographic benchmarks as well as detailed synagogue membership data^1^
US *Jewish Americans in 2020 (2021)*	Pew Research Center, Washington, US	19 November 2019 to 3 June 2020	4,718	Recruitment drawn from a national cross-sectional, address-based sampling (ABS) survey; respondents first identified in a screening survey. Completed online or by mail	Developed using Census Bureau demographic benchmarks, as well as modeled estimates for religious and demographic composition of the eligible respondents

* Synagogue membership data, when subtracted from the estimated total Jewish population size, were used to produce a baseline indicator for affiliation by denomination and non-affiliation.

, 5, 6, 7

**Table 7:** Bivariate analysis of Jewish engagement by country

Variable	Mean	SE	95% CI	
*Jewish ethnocultural capital*^*****^				
Australia	6.5	0.01	6.51	6.56
Canada	5.2	0.04	5.16	5.31
South Africa	6.9	0.02	6.83	6.90
UK	5.6	0.02	5.56	5.66
US	4.8	0.03	4.70	4.83
*Jewish religious capital*^*****^				
Australia	2.4	0.02	2.39	2.47
Canada	2.3	0.03	2.21	2.34
South Africa	3.1	0.03	3.04	3.17
UK	3.2	0.03	3.17	3.30
US	1.9	0.03	1.82	1.92

*Note*: **p* < 0.05; ***p* < 0.01; ****p* < 0.001

, 8

**Table 8:** Games–Howell multiple comparisons test of countries by ethnocultural capital

Country^I^	Country^J^	Mean difference (I − J)	Standard error	Sig	95% confidence interval	
					Lower bound	Upper bound
Australia	Canada	1.30^*^	0.04	0.000	1.18	1.42
	South Africa	−0.338^*^	0.02	0.000	−0.40	−0.28
	UK	0.92^*^	0.03	0.000	0.85	0.10
	US	1.77^*^	0.04	< 0.001	1.67	1.86
Canada	Australia	−1.30^*^	0.04	0.000	−1.42	−1.18
	South Africa	−1.64^*^	0.04	< 0.001	−1.76	−1.52
	UK	−0.38^*^	0.05	< 0.001	−0.51	−0.25
	US	0.47^*^	0.05	0.000	0.33	0.61
South Africa	Australia	0.34^*^	0.02	0.000	0.28	0.40
	Canada	1.64^*^	0.04	< 0.001	1.52	1.76
	UK	1.26^*^	0.03	0.000	1.18	1.34
	US	2.11^*^	0.04	0.000	2.00	2.21
UK	Australia	−0.92^*^	0.03	0.000	−0.10	−0.85
	Canada	0.38^*^	0.05	< 0.001	0.25	0.51
	South Africa	−1.26^*^	0.03	0.000	−1.34	−1.18
	US	0.85^*^	0.04	< 0.001	1.23	1.44
US	Australia	−1.77^*^	0.04	< 0.001	−1.87	−1.67
	Canada	−0.47^*^	0.05	0.000	−0.61	−0.33
	South Africa	−2.11^*^	0.04	0.000	−2.21	−2.01
	UK	−0.85^*^	0.04	< 0.001	−0.96	−0.77

^*^ The mean difference is significant at the 0.05 level

, 9

**Table 9:** Games–Howell multiple comparisons test of countries by religious capital

Country^I^	Country^J^	Mean difference (I − J)	Standard error	Sig	95% confidence interval	
					Lower bound	Upper bound
Australia	Canada	0.16^*^	0.04	< 0.001	0.05	0.27
	South Africa	−0.67^*^	0.04	0.000	−0.77	−0.57
	UK	−0.80^*^	0.04	0.000	−0.90	−0.70
	US	0.56^*^	0.03	< 0.001	0.47	0.65
Canada	Australia	−0.16^*^	0.04	< 0.001	−0.27	−0.05
	South Africa	−0.83^*^	0.05	0.000	−0.96	−0.70
	UK	−0.96^*^	0.05	< 0.000	−1.09	−0.83
	US	0.40^*^	0.04	< 0.001	0.28	0.52
South Africa	Australia	0.67^*^	0.04	0.000	0.57	0.77
	Canada	0.83^*^	0.05	0.000	0.70	0.96
	UK	−0.13^*^	0.05	0.035	−0.25	−0.01
	US	1.23^*^	0.04	0.000	1.12	1.34
UK	Australia	0.80^*^	0.04	0.000	0.70	0.90
	Canada	0.96^*^	0.05	< 0.001	0.83	1.09
	South Africa	0.13^*^	0.05	0.035	0.01	0.25
	US	1.36^*^	0.04	0.000	1.25	1.47
US	Australia	−0.56^*^	0.03	< 0.001	−0.65	−0.47
	Canada	−0.40^*^	0.04	< 0.001	−0.52	−0.28
	South Africa	−1.23^*^	0.04	0.000	−1.34	−1.12
	UK	−1.36^*^	0.04	0.000	−1.47	−1.25

^*^ The mean difference is significant at the 0.05 level

## Footnotes

1 Not all observances and beliefs can, however, be neatly categorized into discrete forms of ethnocultural or religious capital, some observances and beliefs being steeped in dual or alternative significance. Sharot argues that “ethnic Jewish identity can be expressed only through religious symbols and practices” (Sharot 1991, p. 259), but to those individuals and their families, those symbols and practices are devoid of religious intent or salience.

2 For more information on survey methodology, see Graham and Markus (2018), Brym et al. (2019; 2020), Graham (2020), Graham (2021), Graham et al. (2014), and the Pew Research Center (2021).
